# Oral and injectable *Marsdenia tenacissima* extract (MTE) as adjuvant therapy to chemotherapy for gastric cancer: a systematic review

**DOI:** 10.1186/s12906-019-2779-y

**Published:** 2019-12-12

**Authors:** Xu Zhou, Meilu Liu, Qing Ren, Weifeng Zhu, Yang Wang, Haochen Chen, Jianrong Chen

**Affiliations:** 1 Evidence-based Medicine Research Center, Jiangxi University of Traditional Chinese Medicine, Jiangxi, China; 2 School of Chinese Medicine, Hong Kong Baptist University, Hong Kong SAR, China; 3 Second clinical medical college, Chongqing Medical University, Chongqing, China

**Keywords:** MTE, Xiao-ai-ping, Gastric cancer, Chemotherapy

## Abstract

**Background:**

*Marsdenia tenacissima* extract (MTE) is a phytochemical widely used as complementary therapy in cancer care. This systematic review was conducted to investigate the anticancer and detoxification effects of MTE, as an adjuvant therapy to chemotherapy, for treating gastric cancer.

**Methods:**

Ten databases were searched to identify randomized controlled trials (RCTs) comparing oral or injectable MTE plus chemotherapy versus chemotherapy alone for treating gastric cancer up to May 1, 2019. In meta-analyses, proportional odds ratios (PORs) with 95% confidence intervals (CIs) were pooled for the ordinal outcomes using the generalized linear model, and risk ratios (RRs) with 95% CIs were pooled for dichotomous outcomes using the Mantel-Haenszel method.

**Results:**

Seventeen RCTs with 1329 individuals were included, with a moderate to high risk of selection and performance bias. Compared to chemotherapy alone, MTE adjuvant therapy significantly improved the response to anticancer treatment (POR 2.01, 95% CI 1.60–2.53) and patients’ performance status (POR 3.15, 95% CI 2.22–4.48) and reduce the incidences of chemotherapy-induced leukopenia (RR 0.66, 95% CI 0.56–0.78), thrombocytopenia (RR 0.64, 95% CI 0.48–0.86), anemia (RR 0.89, 95% CI 0.72–1.10), nausea/vomiting (RR 0.79, 95% CI 0.69–0.91), hepatic injury (RR 0.77, 95% CI 0.61–0.96), and peripheral neurotoxicity (RR 0.77, 95% CI 0.59–1.01). However, MTE did not significantly alleviate anemia, diarrhea, constipation, kidney injury, and oral mucosal lesions after chemotherapy. Incidence of nausea/vomiting was lower in patients receiving oral MTE than those receiving injectable MTE (RR 0.47 vs. 0.82, interaction *P* = 0.04). Heterogeneity was generally low among these outcomes. Three out of five RCTs that reported survival data supported the effects of MTE for prolonging progression-free and/or overall survival. No studies reported safety outcomes of MTE.

**Conclusions:**

The current evidence with limitations of risk of selection and performance bias suggests that MTE, as an adjuvant therapy to chemotherapy, is effective for inhibiting cancer growth and reducing incidences of multiple chemotherapy side effects. Oral MTE may be a better choice. Uncertainty remains regarding the effects of MTE on survival endpoints and the subgroup differences between acute and chronic use of MTE and between different chemotherapy regimens.

## Background

Gastric cancer is a malignant disease that seriously threatens human health and affects the life expectancy, the global annual incidence of which was approximate 12.1 per 100,000 population [1]. China carries a high burden of gastric cancer which occurred in up to 31.38 per 100,000 people in 2013, representing the third leading cause of cancer-related death (mortality 14.54/100,000) [2]. As a basic treatment, chemotherapy can be used for both patients with early and advanced gastric cancer [3]. However, based on chemotherapy, recurrences still occur in 1.9% of the patients with early gastric cancer after radical gastrectomy [4], and the patients with advanced or recurrent gastric cancer only have a median overall survival (OS) of 20.4 weeks [5]. Moreover, patients who received chemotherapy probably experience toxic side effects, such as gastrointestinal reactions, myelosuppression, and hepatic injury, which substantially reduce patients’ quality of life and even cause life-threaten complications (e.g. acute infections) [6].

Clinicians, therefore, hope to find complementary and alternative approaches for improving the anticancer efficacy and reducing the chemotherapy side effects in the treatment of gastric cancer. Currently, phytochemicals derived from herbal medicine have been developed and used for a complementary and alternative therapy in cancer care worldwide [7]. Multiple phytochemicals have been proven to be effective for anticancer, such as alkaloid, benzopyran, and coumarin [8].

*Marsdenia tenacissima* (family: Apocynaceae) is a representative anticancer herb in traditional Chinese medicine that was initially identified by Lan Mao and documented in *Medicinal Plants in Southern Yunnan* (*Dian Nan Ben Cao*) in Ming Dynasty (600 years ago) [9]. Based on the empirical evidence in several centuries of traditional medicine practice, the stems of *M. tenacissima* is expected to be promising for treating cancer (e.g., lung, esophageal, and gastric cancer) and alleviating chemotherapy-induced adverse effects [10]. In China, the *M. tenacissima* extract (MTE) from the stems has been made into oral or injectable preparations, which is named Xiao-ai-ping [11]. Many animal studies of gastric cancer have revealed that MTE can suppress the growth of cancer cells by inhibiting angiogenesis, eliminating free radicals, and inducting cancer cell apoptosis [12].

Many randomized controlled trials (RCTs) recruiting human subjects evaluated the efficacy of MTE on gastric cancer. The results of these RCTs, however, were inconsistent, which may be attributed to their small sample size and inter-study heterogeneity (e.g., different preparations of MTE [13 14] and different regimens of chemotherapy [15 16]). So far, the effects of MTE as an adjuvant therapy to chemotherapy for treating gastric cancer have not been established. Hence, we conducted a systematic review to inform clinical practice of MTE for gastric cancer by critically assessing and qualitatively synthesizing the current RCT evidence.

## Methods

We reported this systematic review in accordance with the Preferred Reporting Items for Systematic Reviews and Meta-Analyses (PRISMA) statement (Additional file 1) [13].

### Literature search

The relevant literature evidence was searched in ten electronic databases, including PubMed, EMBASE, CENTRAL, ScienceDirect, Scopus, Sinomed, China National Knowledge Internet, Wanfangdata, CQVIP, and Clinicaltrials.gov, from their inception to May 1, 2019. The detailed search strategies in each database are presented in Additional file 2. The reference lists of relevant reviews were also checked for acquiring complementary eligibility.

### Eligible criteria

An eligible study should be an RCT that compared MTE plus chemotherapy versus chemotherapy alone for treating patients with gastric cancer and reported data on at least one of the outcomes of interest. The patients should be diagnosed as gastric cancer by histopathological examination. No restrictions were imposed on publication language, stage of gastric cancer, regimen of treatment, and length of follow up. We excluded studies that used any other traditional Chinese medicine in either group, assessed outcomes using unclear standards, or had insufficient data for data analysis.

### Outcomes

We assessed the following outcomes of interest:

1. Response to treatment assessed by the Response Evaluation Criteria in Solid Tumors (RECIST) version 1.1 [14]. The response to treatment was graded as four ranks: 1) complete response (CR): all target lesions disappeared; 2) partial response (PR): the total dimension of the target lesions decreased by 30% or more compared with baseline; 3) progressive disease (PD): new lesions developed or the total dimension of the target lesions increased by 20% or more; 4) stable disease (SD): changes in the target lesions did not meet any of the above criteria.
2. Performance status assessed by the Karnofsky Performance Status Scale [15]. The patients’ performance status was divided into ten levels, including normal (100%), minor symptoms (90%), some symptoms (80%), unable to do active work (70%), occasionally requires assistance (60%), usually requires assistance (50%), disabled (40%), severely disabled (30%), admission to hospital (20%), moribund (10%), and dead (0%). The performance status improved ≥1 level, did not change, and declined ≥1 level were considered to be “improved”, “stable” and “deteriorated”.
3. Chemotherapy side effects whose severity was classified as grade 1 or more according to the World Health Organization criteria [16]. We assessed the following side effects: myelosuppression (leukopenia, thrombocytopenia, and anemia), gastrointestinal reactions (nausea/vomiting, diarrhea, and constipation), hepatic injury, kidney injury, peripheral neurotoxicity, and oral mucosal lesions.
4. PFS and OS.
5. Safety of MTE assessed by incidence of MTE-related adverse effects.

### Study selection and data extraction

Two reviewers, in pairs and independently, read titles and abstracts to identify preliminarily eligible studies and then read full texts to determine the final inclusions. The following data of the included studies was extracted using a standardized form with a pilot test: title, author, publication year, gender, mean age, stage of gastric cancer, preparation of MTE, regimen of chemotherapy, course of treatment, length of follow-up, and outcome data. Any disagreements were settled by inter-reviewer discussion or consultation with a third reviewer.

### Risk of bias assessment

We evaluated the following domains of bias for each RCT referring to the Cochrane risk of bias tool [17]: 1) selection bias (inappropriate random sequence generation and allocation concealment); 2) performance bias (unblinded patients and clinicians); 3) detection bias (unblinded outcome assessors); 4) attrition bias (incomplete outcome data); 5) reporting bias (selective reporting of outcomes); and 6) other bias (e.g., imbalanced baseline characteristics). Each domain was judged to be at low, high, or unclear risk. Two reviewers assessed the risk of bias independently and in duplicate and crosschecked the results. Any discrepancies were addressed by inter-reviewer discussion or consultation with a third reviewer.

### Data analysis

Frequency and incidence were used as the descriptive statistics for all outcomes. The dichotomous outcomes were measured by risk ratios (RRs) with 95% confidence intervals (CIs) and pooled by the Mantel-Haenszel method. The ordinal outcomes (i.e., response to treatment and performance status) were measured by proportional odds ratios (PORs) with 95% CIs. We first calculated the natural logarithm of the POR (logPOR) and its standard error (selogPOR) for each RCT using the generalized linear model and then pooled the individual results by the generic inverse variance method [18].

Heterogeneity across studies was quantitatively assessed by the I^2^ statistics, and an I^2^ > 50% indicated a significant heterogeneity. Given that there were always clinical and methodological varieties among the included RCTs, we performed all meta-analyses under a random effects model. To explore the cause of heterogeneity, we performed a set of subgroup analyses stratified by the different preparations of MTE (oral versus injectable). An interaction *P* < 0.05 indicated a significant between-subgroup difference. Funnel plots and Egger’s tests were used to examine the presence of publication bias for each outcome. Duval and Tweedie’s trim and fill test was used to adjust the results with significant publication bias [19]. SAS v9.4 (SAS Institute Inc., NC, USA) was used to calculate the PORs and perform the tests for publication bias; RevMan v5.3.5 (St. Louis, Missouri, USA) was used to perform the meta-analyses and draw the funnel plots.

### Level of evidence assessment

The Grading of Recommendations Assessment, Development and Evaluation (GRADE) instrument was used for assessing the level of evidence for the outcomes with meta-analytic result. Five aspects of limitation of evidence were assessed, including risk of bias, imprecision, inconsistency, indirectness, and publication bias.

## Results

### Study description

**Fig. 1:**
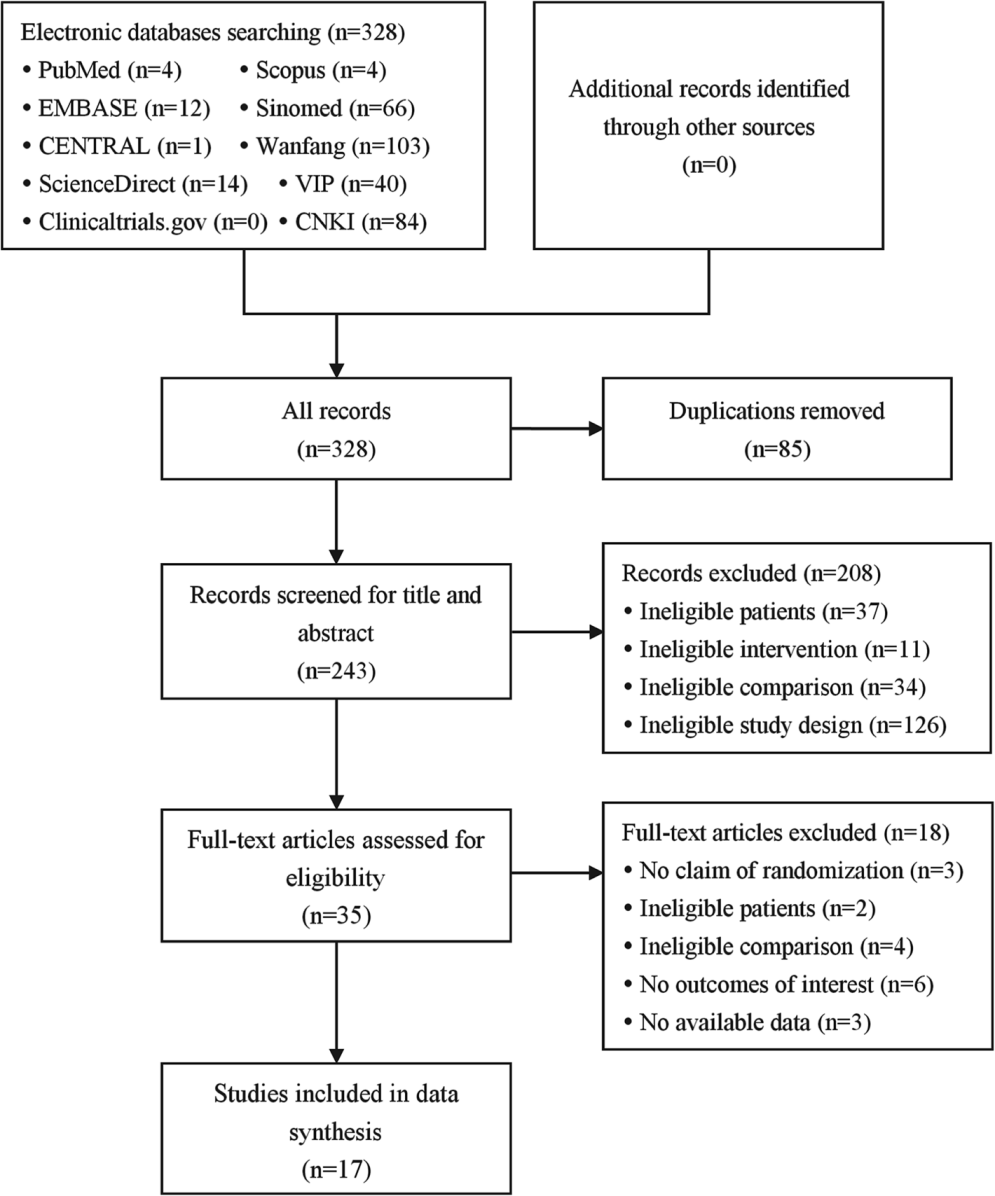
Flowchart of study screening

The literature search identified 328 records, and 17 RCTs [13–16 24–36] involving 1329 patients were finally included after the screening (Fig. 1). Among the included RCTs, the overall proportion of male was 55.9% and the mean age ranged from 51.5 to 68.2 years. Two trials recruited patients with early gastric cancer and the rest recruited patients with advanced gastric cancer. Injectable MTE was used in 13 trials and oral MTE in 4 trials. MTE was administered as an acute treatment (injectable MTE: 40–80 ml/d, 7–21 d/session, 2–4 sessions; oral MTE: 6–7.2 g/d, 30 d/session, 2 sessions) along with the chemotherapy in all trials. The most common chemotherapy regimen was FOLFOX (folinic acid + fluorouracil + oxaliplatin, 5 trials), followed by XELOX (capecitabine + oxaliplatin, 4 trials). Table 1 presents the study characteristics in details.

**Table 1:** Characteristics of included randomized controlled trials

Study	No. of patients		Stage of gastric cancer	MTE (type and dose)	Chemotherapy regimen for both groups	Co-intervention for side effects	Length of follow up (week)	Outcome
	MTE group	Control group						
Deng 2016 [20]	15	15	Advanced	MTE injection, 80 ml/d for 7 d, 2 sessions	TP	5-HT3 antagonists	6	a,b,c
Gao 2015 [21]	92	91	Advanced	MTE injection, 40 ml/d for 14 d, 2 sessions	XELOX	Tropisetron and CSF	16	a,c,d,e,g
Gao 2017 [22]	33	33	IV	MTE injection, 80 ml/d for 14 d, 4 sessions	TP	NR	12	a,b,c,d,e,g
Huang 2013 [23]	36	36	Early stage with radical gastrectomy	MTE tablet, 2.4 g/time, 3 times/d for 30 d, 2 sessions	FOIFOX/XELOX/EOF	NR	26	c,d,
Huo 2009 [24]	31	31	Advanced	MTE tablet, 2.4 g/time, 3 times/d for 30 d, 2 sessions	FOIFOX	Granisetron	8	a
Keyoumu 2012 [25]	33	35	III and IV	MTE injection, 60 ml/d for 7 d, 2 sessions	FOLFOX	Ondansetron	14.2	a,b,c,d,e,g,h
Li 2016 [26]	60	60	IV	MTE injection, 80 ml/d for 14 d, 4 sessions	CPT-11	NR	8	a,c,e,g,h,i
Lin 2015 [27]	28	28	Advanced	MTE injection, 60 ml/d for 14 d, 2 sessions	XELOX	Tropisetron, metoclopramide, vitamin B6, and CSF	6	a,b,c,d,e,f,g,i
Liu 2012 [28]	28	28	IV	MTE injection, 80 ml/d for 7 d, 4 sessions	FOLFOX	5-HT3 antagonists	8	a,c,d,e,f,g
Liu 2017 [29]	48	48	III and IV	MTE injection, 60 ml/d for 14 d, 2 sessions	SOX	Palonosetron and metoclopramide	12	a,b,c,d,g,h,i
Ma 2015 [30]	23	23	Advanced	MTE injection, 60 ml/d for 7 d, 2 sessions	SOX	NR	6	b,c
Shi 2017 [31]	53	53	Early stage with radical gastrectomy	MTE tablet, 2.4 g/time, 3 times/d for 30 d, 2 sessions	EOF/OLF	NR	26	c,d,
Xiong 2015 [32]	32	32	IV	MTE injection, 80 ml/d for 21 d, 4 sessions	chemotherapy	Vitamin	12	a,g,i,j
Zhang HY 2015 [33]	46	46	III and IV	MTE capsule, 2.0 g/time, 2 times/d for 30 d, 2 sessions	PF	NR	8	a,b,c,d
Zhang H 2015 [34]	25	23	Advanced	MTE injection, 40 ml/d for 14 d, 2 sessions	XELOX	CSF	6	a,b,c,d,e,f,g,h
Zheng 2017 [35]	42	42	III and IV	MTE injection, 60 ml/d for 21 d, 2 sessions	chemotherapy	NR	18	a,c,d,e,g,h,i,j
Zhu 2017 [36]	40	40	Advanced	MTE injection, 60 ml/d for 14 d, 4 sessions	FOLFOX	Tropisetron	8	a,c,d,e,g,h

Abbreviations: *T* trial group; *C* control group; *MTE Marsdenia tenacissima* extract; *NR* not reported; *FOLFOX* folinic acid + fluorouracil + oxaliplatin; *XELOX* capecitabine + oxaliplatin; *TP* docetaxel + cisplatin; *SOX* S-1 + oxaliplatin; *EOF* epirubicin + oxaliplatin + fluorouracil; *OLF* oxaliplatin + leucovorin + flurouracil; *CPT-11* irinotecan; *PF* cabazitaxel + Platinum + fluorouracil; *CSF* colony stimulating factor

Outcomes: a = response to treatment; b = performance status; c = myelosuppression; d = gastrointestinal reactions; e = hepatic injury; f = kidney injury; g = neurotoxicity; h = oral mucosal lesions; i = progression-free survival; j = overall survival

### Risk of bias

**Fig. 2:**
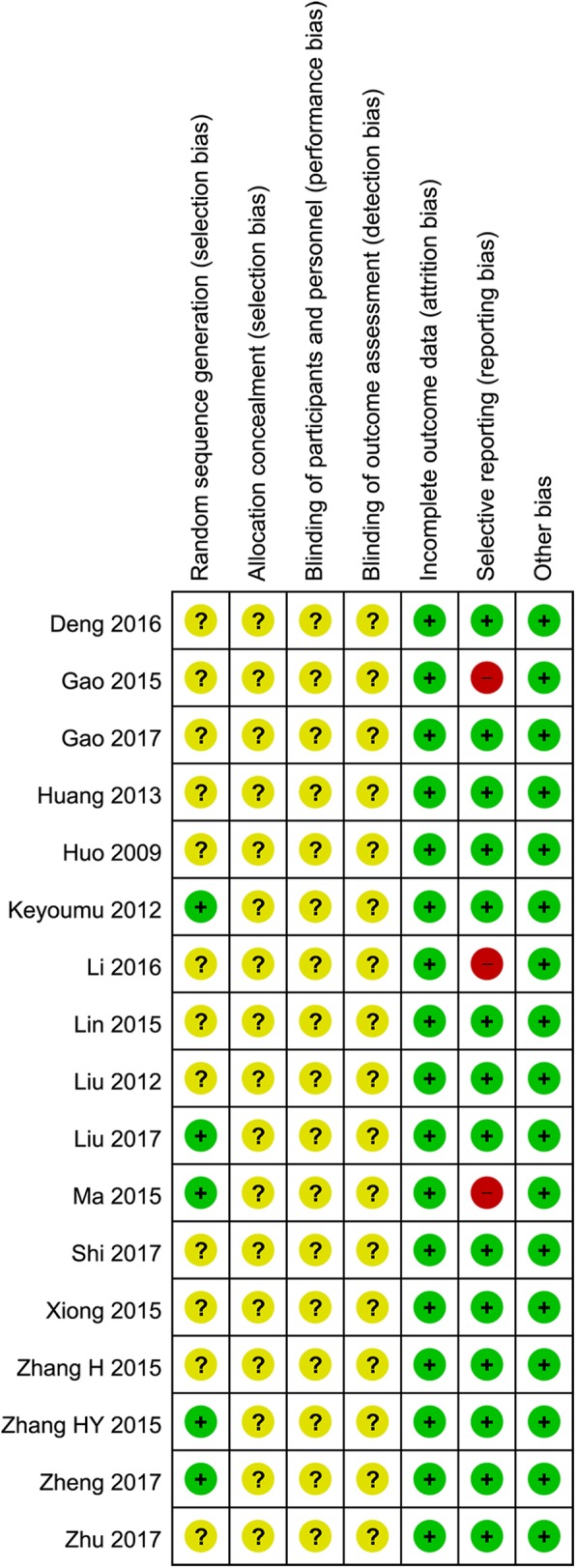
Risk of bias assessment. Note: The symbols “+”, “-”, and “?” indicate low, high, and unclear risk of bias, respectively

As shown in Fig. 2, all RCTs were considered to be at a moderate to high risk of bias. Specifically, five RCTs [27, 31, 32, 34, 35] generated the allocation sequence using a random number table, and the others did not report the method of allocation sequence generation. No RCTs reported information on allocation concealment and blinding of patients, clinicians, and outcome assessors. Four RCTs [26, 29, 32, 35] lost a few (1 to 6) patients during the follow-up, while the others had a complete follow-up. Three RCTs [24, 28, 32] seemed to have selective reporting since they did not report all planned outcomes or did not provide sufficient outcome data.

### MTE in adjutant with chemotherapy for management of gastric cancer

#### Response to treatment

**Fig. 3:**
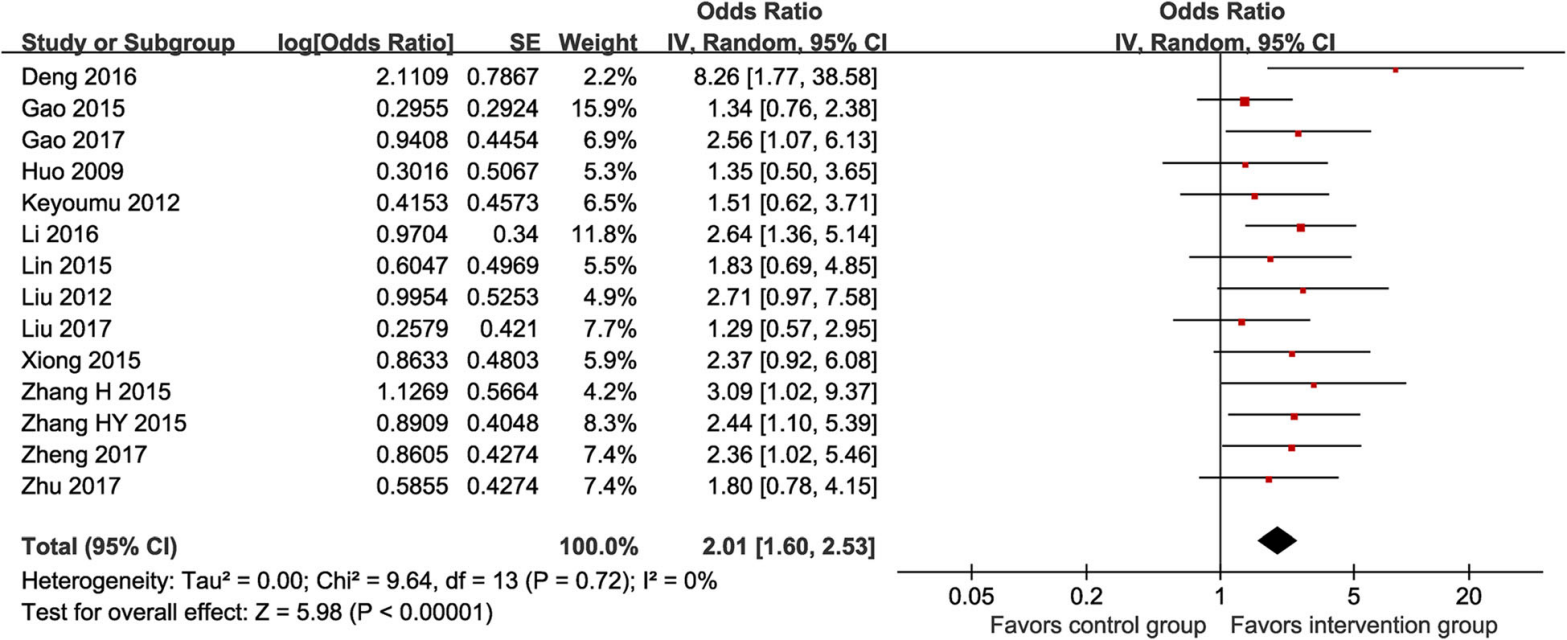
Meta-analysis on response to treatment

Fourteen RCTs (*n* = 1079) [14–16, 24, 26–31, 33–36] reported data on the response to treatment assessed by the RECIST v1.1 criteria. In total, the MTE group had 57 cases of CR (10.6%), 251 PR (46.8%), 158 SD (29.5%), and 70 PD (13.1%) and the control group had 34 CR (6.3%), 191 PR (35.2%), 168 SD (30.9%), and 150 PD (27.6%). As shown in Fig. 3, the ordinal data meta-analysis showed that the MTE group had a significantly higher probability of improving more than one rank in the response to treatment than the control group (POR 2.01, 95% CI 1.60–2.53, I^2^ = 0%).

#### Performance status

**Fig. 4:**
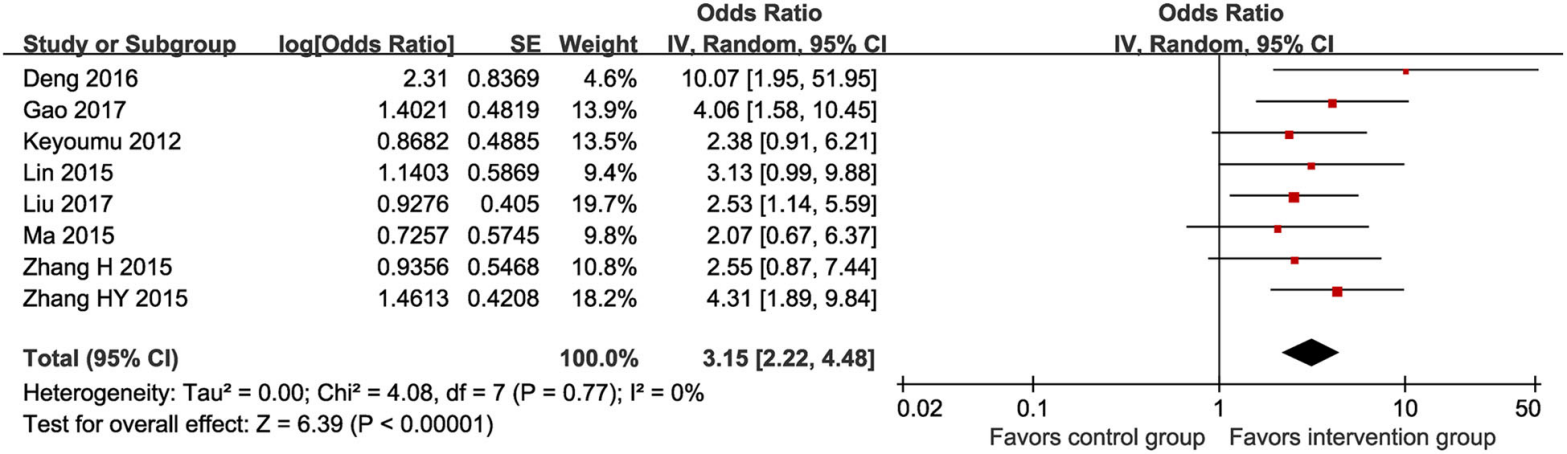
Meta-analysis on performance status

Eight RCTs (*n* = 502) [15, 24, 27, 29, 31–34] described data on the performance status assessed by the Karnofsky scale. The number of patients who had improved, stable, and deteriorated performance status was 151 (60.6%), 73 (29.3%), and 25 (10.0%) in the MTE group and 76 (30.0%), 96 (37.9%), and 81 (32.0%) in the control group, respectively. The MTE group showed significantly more improvement in the performance status than the control group (POR 3.15, 95% CI 2.22–4.48, I^2^ = 0%; Fig. 4).

### Progression-free and overall survival

Five RCTs [14, 28, 29, 31, 35] and two RCTs [14, 35] assessed PFS and OS, respectively. Because of insufficient reporting of parameters, meta-analyses were not available for these outcomes. Three out of five RCTs (*n* = 420) reported that the MTE group had a significantly longer PFS than the control group (median PFS: 10.48 vs. 9.48 months in *Li 2016*, *P* < 0.05 [26]; 8.41 vs. 6.01 months in *Xiong 2015*, *P* < 0.05 [32]; 6.3 vs. 5.4 in *Zheng 2017*, *P* < 0.05 [35]), but the remaining two did not find such difference (5.57 vs. 5.50 months in *Lin 2015*, *P* > 0.05 [27]; 7.0 vs. 6.5 months in *Liu 2017*, *P* = 0.746 [29]). Both RCTs (*n* = 148) that assessed OS reported that the MTE group had a significantly longer OS than the control group (median OS: 10.36 vs. 8.62 months in *Xiong 2015*, *P* < 0.05 [32]; 9.6 vs. 8.0 months in *Zheng 2017*, P < 0.05 [35]).

### MTE for reducing adverse effect of chemotherapy

#### Myelosuppression

**Fig. 5:**
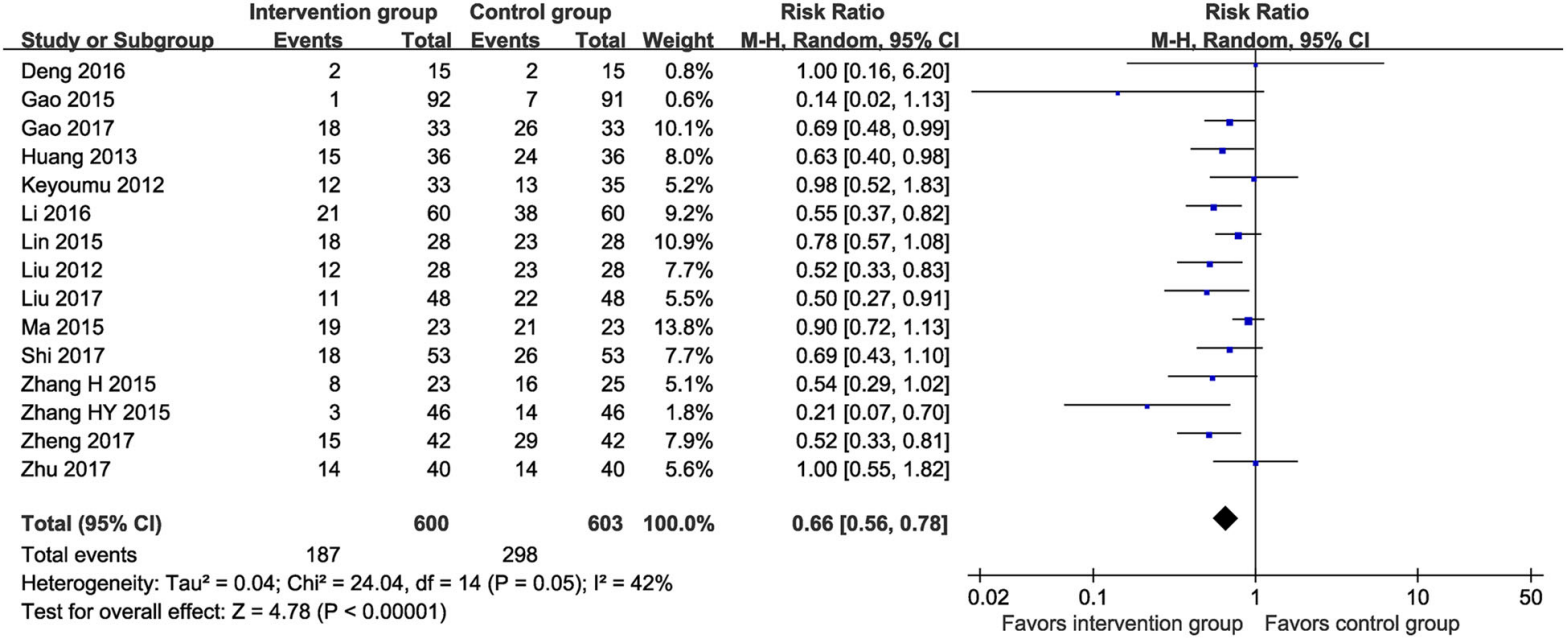
Meta-analysis on incidence of leukopenia

Fifteen RCTs (*n* = 1203) [13, 15, 16, 24, 25, 27–36] reported data on the incidence of leukopenia (MTE vs. control: 31.2% vs. 49.4%). The meta-analysis found a significantly lower incidence of leukopenia in the MTE group than the control group (RR 0.66, 95% CI 0.56–0.78, I^2^ = 42%; Fig. 5).

**Fig. 6:**
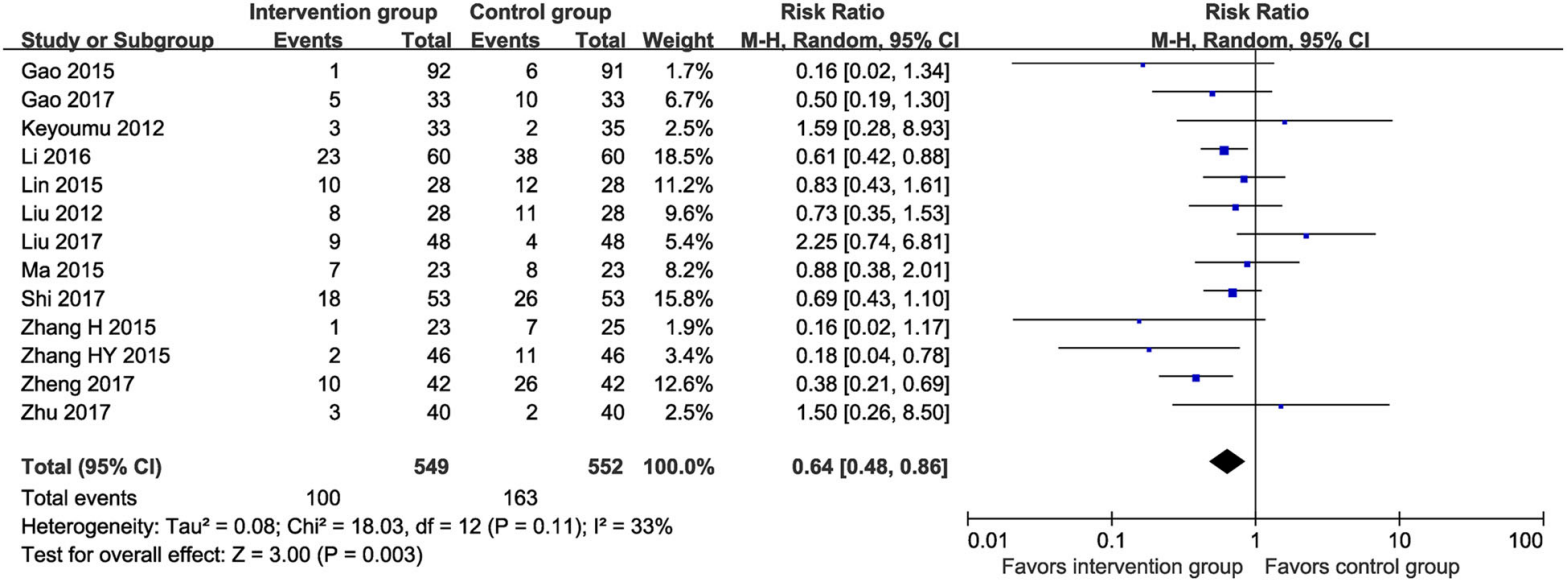
Meta-analysis on incidence of thrombocytopenia

Thirteen RCTs (*n* = 1101) [13, 16, 24, 27–36] reported data on the incidence of thrombocytopenia (MTE vs. control: 18.2% vs. 29.5%). The meta-analysis found the incidence of thrombocytopenia was significantly lower in the MTE group than the control group (RR 0.64, 95% CI 0.48–0.86, I^2^ = 33%; Fig. 6).

**Fig. 7:**
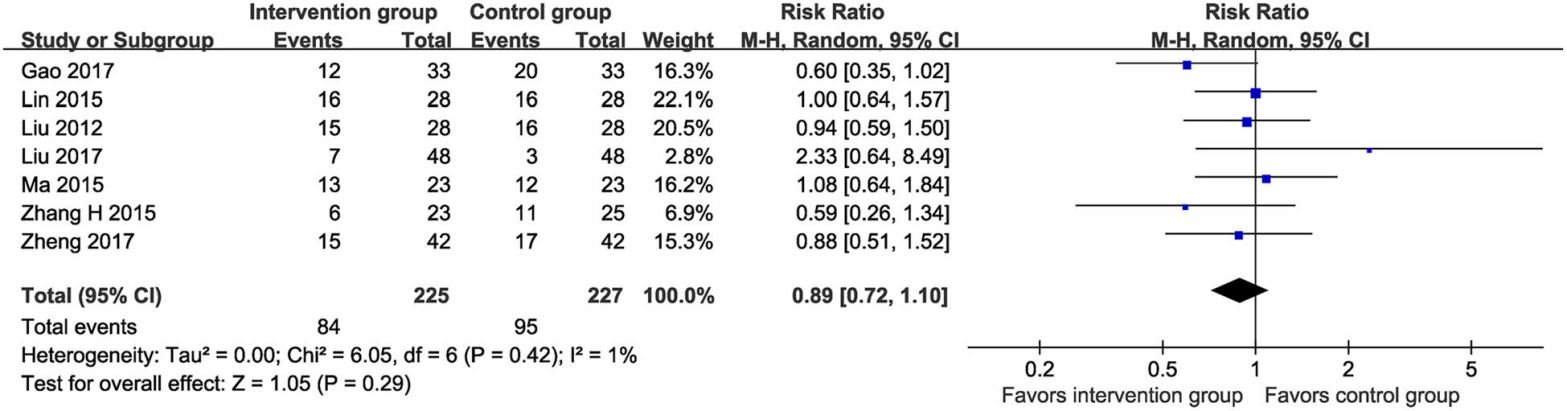
Meta-analysis on incidence of anemia

Seven RCTs (*n* = 452) [24, 29–33, 35] reported data on the incidence of anemia (MTE vs. control: 37.3% vs. 41.9%). The meta-analysis suggested no significant difference in the incidence of anemia between the two groups (RR 0.89, 95% CI 0.72–1.10, I^2^ = 1%; Fig. 7).

#### Gastrointestinal reactions

**Fig. 8:**
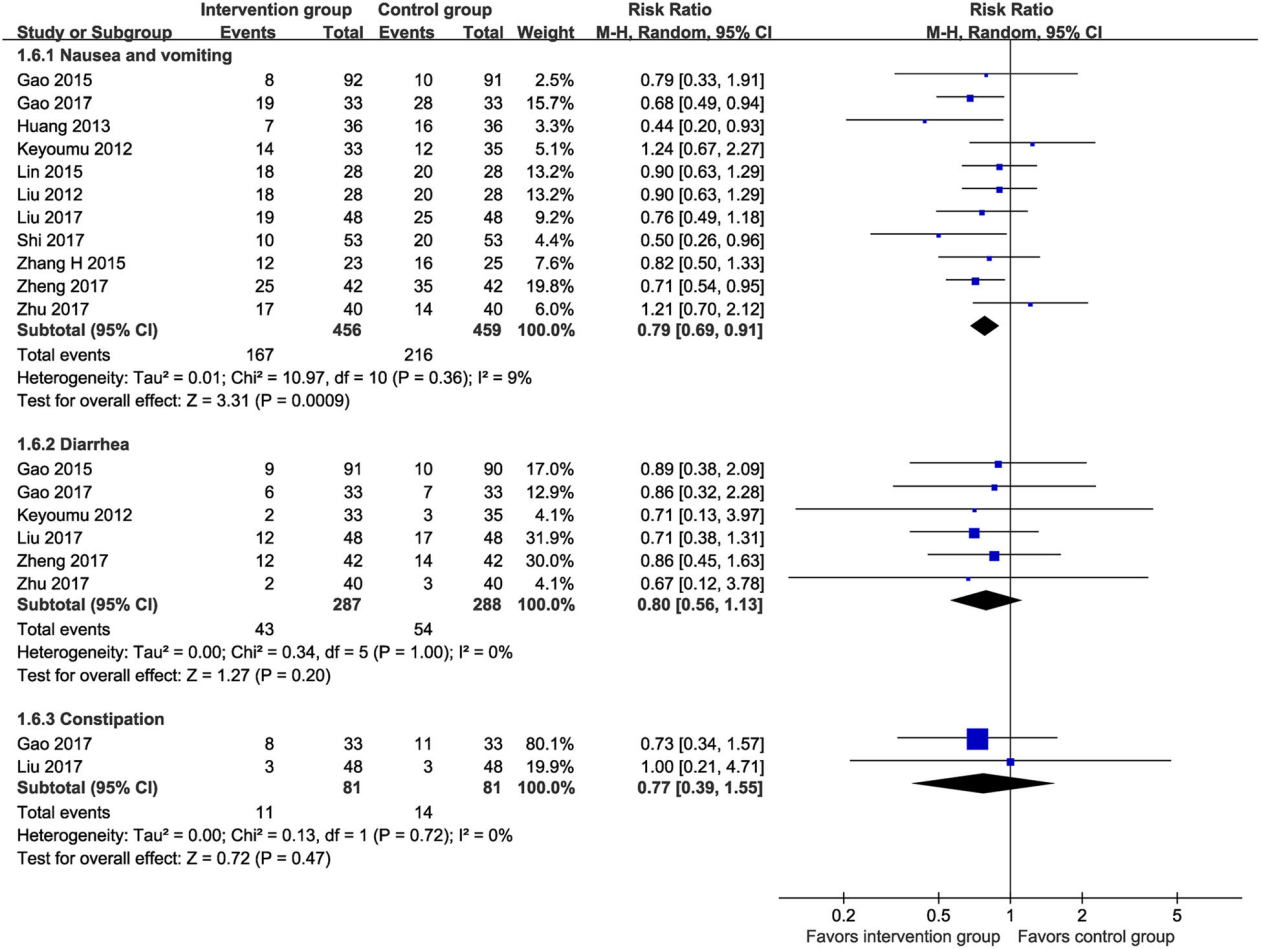
Meta-analysis on incidence of gastrointestinal reactions

Data on nausea/vomiting, diarrhea, and constipation were reported in eleven RCTs (*n* = 915) [13, 16, 24, 25, 27, 29–31, 33, 35, 36], six RCTs (*n* = 575) [16, 24, 27, 31, 35, 36], and two RCTs (*n* = 162) [24, 31], respectively. As shown in Fig. 8, the meta-analyses showed a significantly lower incidence of nausea/vomiting in the MTE group than the control group (36.6% vs. 47.1%; RR 0.79, 95% CI 0.69–0.91, I^2^ = 9%) but failed to show favorable results for the MTE group on diarrhea (15.0% vs. 18.8%; RR 0.80, 95% CI 0.56–1.13, I^2^ = 0%) and constipation (13.6% vs. 18.8%; RR 0.77, 95% CI 0.39–1.55, I^2^ = 0%).

#### Other side effects

**Fig. 9:**
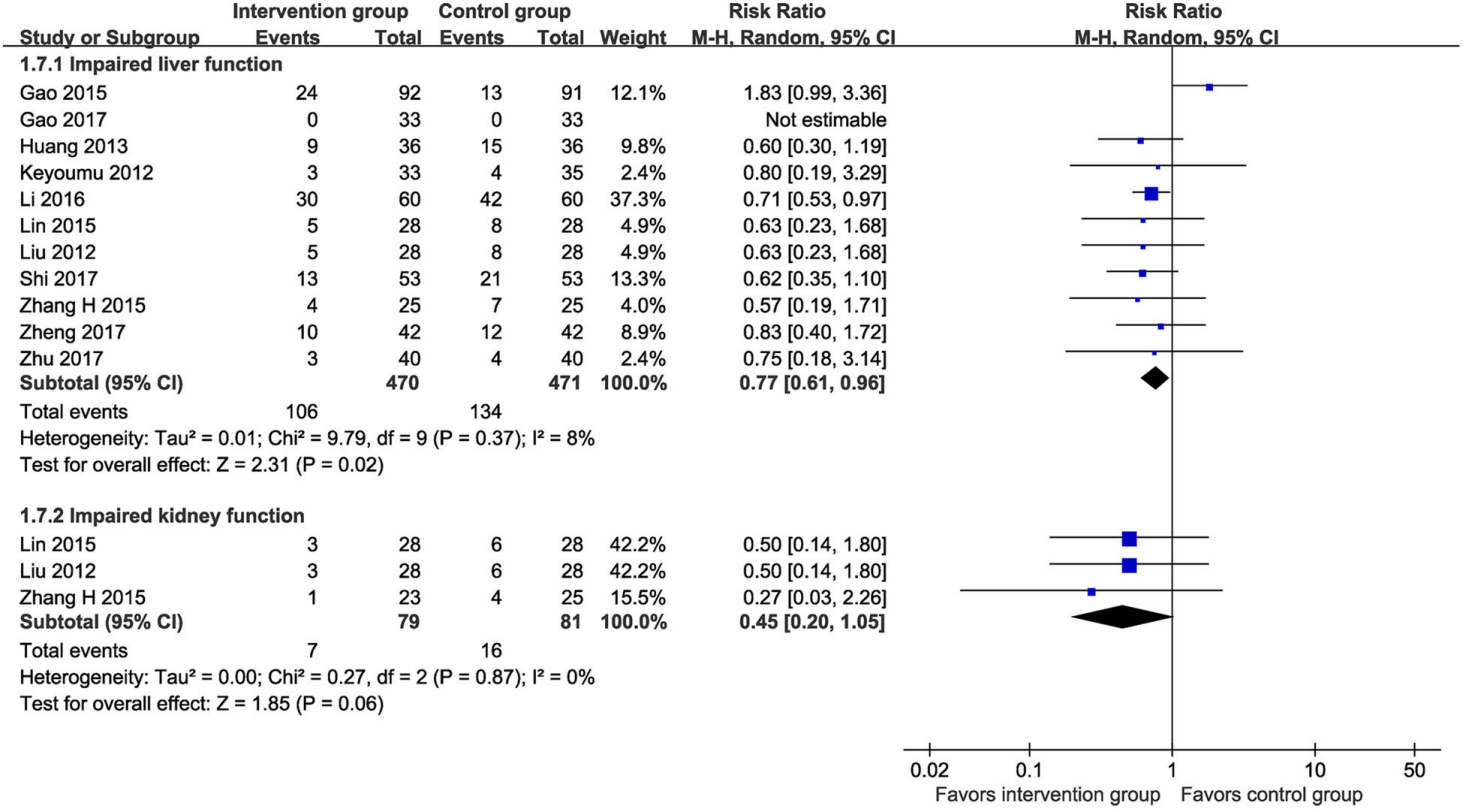
Meta-analysis on incidence of hepatic and kidney injury

Eleven RCTs (*n* = 941) [13, 16, 24, 25, 27–30, 33, 35, 36] and three RCTs (*n* = 160) [29 31 33] reported data on hepatic injury and kidney injury after chemotherapy, respectively. As shown in Fig. 9, the MTE group had a significantly less frequency of hepatic injury (22.6% vs. 28.5%; RR 0.77, 95% CI 0.61–0.96, I^2^ = 8%) but a comparable frequency of kidney injury compared with the control group (8.9% vs. 19.8%; RR 0.45, 95% CI 0.20–1.05, I^2^ = 0%).

**Fig. 10:**
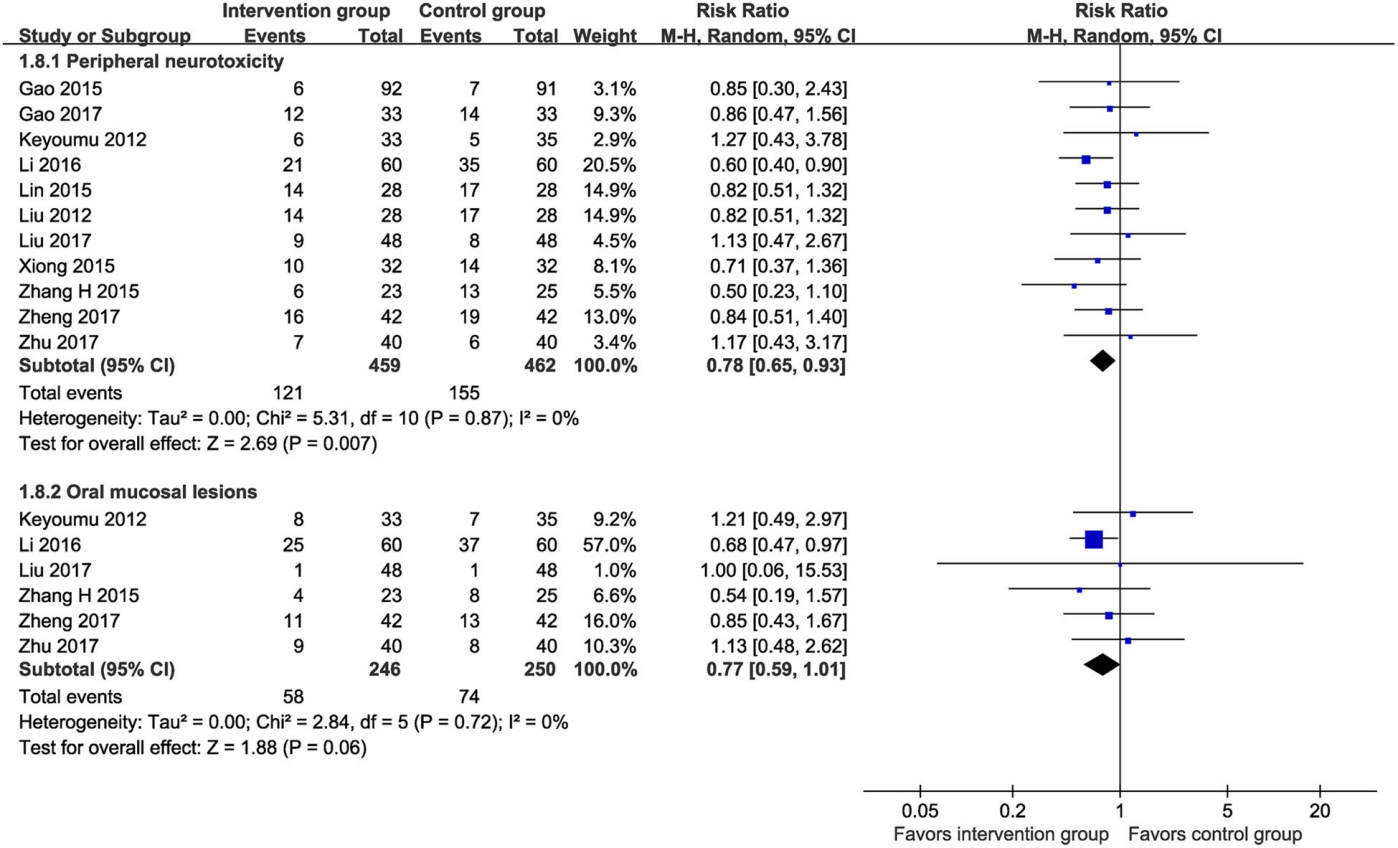
Meta-analysis on incidence of peripheral neurotoxicity and oral mucosal lesions

Eleven RCTs (*n* = 921) [14, 16, 24, 27–31, 33, 35, 36] tested the effects of MTE on reducing peripheral neurotoxicity induced by chemotherapy. The incidence of peripheral neurotoxicity was 26.4% in the MTE group and 33.6% in the control group. The meta-analysis suggested a favorable effect of MTE on reducing the incidence of peripheral neurotoxicity (RR 0.78, 95% CI 0.65–0.93, I^2^ = 0%; Fig. 10).

Six RCTs (*n* = 496) [27, 28, 31, 33, 35, 36] tested the effects of MTE on oral mucosal lesions. The incidence of oral mucosal lesions was 23.6% in the MTE group and 29.6% in the control group, without a significant between-group difference (RR 0.77, 95% CI 0.59–1.01, I^2^ = 0%; Fig. 10).

### Safety

No studies reported safety information regarding MTE.

### Subgroup analysis

The results of subgroup analyses stratified by the different preparations of MTE are presented in Table 2. Patients who received oral MTE had a significantly lower incidence of nausea/vomiting compared with those who received injectable MTE (RR 0.47 vs. 0.82, interaction *P* = 0.04). No significant subgroup difference was found for other outcomes, indicated by an interaction *P* > 0.05.

**Table 2:** Subgroup analysis stratified by injectable and oral *Marsdenia tenacissima* extract

Outcome	Injectable MTE subgroup			Oral MTE subgroup			Interaction *P* value
	No. of studies	Estimate (95% CI)	I^2^	No. of studies	Estimate (95% CI)	I^2^	
Response to treatment	12	POR 2.02 (1.58–2.58)	0%	2	POR 1.94 (1.04–3.60)	0%	0.90
Performance status	7	POR 2.94 (1.99–4.34)	0%	1	POR 4.31 (1.89–9.84)	0%	0.41
Leukopenia	12	RR 0.68 (0.57–0.82)	42%	3	RR 0.57 (0.36–0.90)	44%	0.48
Thrombocytopenia	11	RR 0.67 (0.49–0.93)	33%	2	RR 0.42 (0.11–1.59)	69%	0.50
Nausea/vomiting	9	RR 0.82 (0.71–0.94)	0%	2	RR 0.47 (0.29–0.78)	0%	0.04
Hepatic injury	9	RR 0.83 (0.62–1.12)	19%	2	RR 0.61 (0.39–0.95)	0%	0.25

Note: Subgroup analysis was unavailable for anemia, diarrhea, constipation, kidney injury, peripheral neurotoxicity, and oral mucosal lesions because all trials that assessed these outcomes used injectable *Marsdenia tenacissima* extract

Abbreviations: *MTE Marsdenia tenacissima* extract; *POR* proportional odds ratio; *RR* risk ratio; *CI* confidence interval

### Publication bias

Based on the results of the funnel plots and Egger’s tests, publication bias was considered to be significant for the response to treatment (Egger’s test *P* = 0.037) but not significant for the incidences of leukopenia (*P* = 0.225), thrombocytopenia (*P* = 0.778), nausea/vomiting (*P* = 0.971), hepatic injury (*P* = 0.466), and peripheral neurotoxicity (*P* = 0.121). The adjusted analysis using the trim and fill tests for the response to treatment did not show apparent changes (POR 1.90, 95% CI 1.50–2.42). The tests for publication bias were not available for the other outcomes due to insufficient sample size.

### Level of evidence

The level of evidence assessment showed that all outcomes did not suffer serious limitation on inconsistency, indirectness, and publication bias but suffered serious to very serious limitation on risk of bias and/or imprecision. As a result, four (leukopenia, thrombocytopenia, nausea/vomiting, peripheral neurotoxicity), six (response to treatment, performance status, anemia, diarrhea, hepatic injury, and oral mucosal lesions), and two outcomes (constipation and kidney injury) were judged as moderate, low, and very low level of evidence, respectively (Table 3).

**Table 3:** Level of evidence assessment using GRADE approach for the outcomes

Study	Risk of bias	Imprecision	Inconsistency	Indirectness	Publication bias	Level of evidence
*Marsdenia tenacissima* extract in adjutant with chemotherapy for management of gastric cancer						
Response to treatment	-1	−0	− 0	− 0	− 1	Low
Performance status	−1	− 1	− 0	− 0	− 0	Low
*Marsdenia tenacissima* extract for reducing adverse effect of chemotherapy						
Leukopenia	−1	−0	− 0	− 0	− 0	Moderate
Thrombocytopenia	− 1	− 0	− 0	− 0	− 0	Moderate
Anemia	−1	− 1	− 0	− 0	−0	Low
Nausea/vomiting	− 1	−0	−0	− 0	−0	Moderate
Diarrhea	−1	− 1	−0	−0	− 0	Low
Constipation	−1	−2	−0	−0	− 0	Very low
Hepatic injury	−1	−0	−0	− 0	−0	Low
Kidney injury	−2	− 2	−0	−0	− 0	Very low
Peripheral neurotoxicity	−1	−0	−0	− 0	−0	Moderate
Oral mucosal lesions	−1	− 1	−0	−0	− 0	Low

Note: In GRADE instrument, the level of evidence of outcomes is judged as high, moderate, low, and very low if the total score is “-0”, “-1”, “-2”, and “≥ −3”, respectively

## Discussion

This systematic review was conducted to evaluate the effectiveness of MTE against gastric cancer. The results revealed that MTE, as an adjuvant therapy to chemotherapy, improved the response to anticancer treatment and patients’ performance status, and meanwhile, reduced the incidences of leukopenia, thrombocytopenia, nausea/vomiting, hepatic injury, and peripheral neurotoxicity induced by chemotherapy. However, MTE did not significantly alleviate anemia, diarrhea, constipation, kidney injury, and oral mucosal lesion after chemotherapy. The effects of MTE on PFS and OS were uncertain.

The response to treatment was assessed by the RECIST criteria, which focused on the changes in the dimension of cancer lesions. Hence, the findings on the response to treatment imply that MTE could inhibit the growth of gastric cancer cells. Based on the current in vitro and animal research evidence, phenolic acid, C-21 steroidal glycosides, and polyphenols in MTE may play a critical role in its anticancer mechanism, which could suppress angiogenesis in cancer tissues by blocking the activation of vascular endothelial growth factor receptors and phosphorylated protein kinases [12, 37, 38]. MTE also has effects of prolonging mitosis cycle and inducing apoptosis for cancer cells, which may be modulated by multiple factors, such as phosphoinositide 3-kinases, protein kinase B, mammalian target of rapamycin, and extracellular regulated protein kinases [39].

The incidences of multiple chemotherapy side effects were reduced after the MTE treatment, especially for thrombocytopenia (− 36%) and leukopenia (− 34%), with acceptable heterogeneity. Similar effects of MTE for reducing the adverse effects of chemotherapy have been reported in previous research [39, 40], but the underlying mechanism is unclear since there is a lack of relevant pharmacological studies. The subgroup analysis suggested a favorable effect on relieving nausea/vomiting for the oral MTE compared with the injectable MTE. Given that there was comparable efficacy on the response to treatment and performance status between the oral and injectable preparations, oral MTE appears to be a better choice. However, this finding needs further evidence because the subgroup analysis included only two RCTs of oral MTE.

In cancer research, PFS and OS are both important long-term endpoints. Although significant anticancer effects were found for the surrogate outcomes (i.e., the response to anticancer treatment and performance status) in our review, whether MTE will ultimately prolong PFS and OS still lacks evidence. The results of PFS were inconsistent across the included RCTs; and for OS, the sample size (74 in each group) is too small to yield a definite conclusion. Moreover, even if that the between-group difference was statistically significant, the absolute estimates (mean difference of survival time) would be only approximately one month for median PFS and two months for median OS based on the reported data, the clinical implication of which might be limited.

The safety of herbal extracts has been a target of public criticism [41]. We were unable to assess the safety of MTE because no such information was reported. Currently, there are no reports of acute or subacute toxicity of oral MTE. A rat study reported that oral MTE did not cause any toxicity effects or outcomes at an acute toxicity dose of 5 g/kg body weight for 14 days and subacute doses of 0.25, 0.5, and 1 g/kg body weight for 28 days [42]. Another rat study also showed that an acute oral dose of 2 g/kg body weight MTE did not cause any deaths up to 2 days [43]. However, some observational human studies have reported a number of adverse events in malignant patients caused by MTE, such as rash, shiver, chills, malaise, nausea, abdominal pain, and palpitation, all of which were determined to be associated with MTE by the Provincial Food and Drug Administrations and most of which occurred within 60 min after the administration and when MTE was administered as an injection [44, 45]. Therefore, the administration of MTE—injection in particular—needs a close observation of patients’ adverse reactions during the medication.

A previous systematic review has assessed the effect of MTE on gastric cancer [46]. However, the review was only focused on the injectable MTE and advanced gastric cancer, assessed less chemotherapy-induced side effect outcomes, and suffered some methodological limitations in its data analysis of primary outcomes. For example, its conclusions were predominately drawn from the subgroup analyses stratified by different chemotherapy regimens, but the subgroup analyses did not include all regimens, which was an incorrect way. The results of tests for subgroup difference (i.e., interaction *p* values) were also not considered in the interpretation of the subgroup effects. These limitations finally misled the subgroup findings -- the review found that the anticancer effects of injectable MTE were significant in patients receiving XELOX but not significant in patients receiving FOLFOX and S-1 + oxaliplatin in both main outcomes, while these subgroup differences were actually false-positive that can be explained by chance because the interaction p values were 0.40 and 0.78 (> 0.05), respectively [47]. Furthermore, the review had no appraisal on the quality of evidence for the outcomes.

Compared with the previous review, our systematic review included additional four RCTs, assessed and compared oral and injectable MTE, and reported more chemotherapy-induced side effect outcomes, including thrombocytopenia, anemia, diarrhea, constipation, peripheral neurotoxicity, and oral mucosal lesions. Our systematic review also has several strengths in methodology. First, we defined consistent criteria (i.e., the RECIST criteria and the Karnofsky scale) to assess the response to treatment and performance status, which facilitated lowering the heterogeneity and interpreting the results. Second, the previous review used an inappropriate method to analyze the ordinal variables in which the ordinal variables were converted to be dichotomous by combining adjacent values using a cut-point. This method lost the difference within the combined values and may bias the results [45, 48]. Oppositely, we calculated the PORs using the generalized linear model by assuming that the odds ratios were proportional for all dichotomies of the values, which can maximize the information utilization and yield more reasonable results. Third, the overall heterogeneity was low in the meta-analyses, and part of it was explained by the subgroup finding (oral versus injectable MTE) with an interaction *p* value less than 0.05—the reliability of the relevant results was thus improved. Fourth, we critically appraised the quality of evidence for each outcome using the GRADE instrument, which increased the precision and applicability of the findings for the clinical practice and guideline development.

There are some limitations in this review due to the inherent deficiency of the included RCTs. First, because all RCTs were at a high risk of selection and performance bias, the results may be impacted by inadequate randomization and placebo effects, which substantially weakens the level of evidence of all outcomes. Second, acute or chronic use of MTE and different chemotherapy regimens may be important causes of heterogeneity. However, we did not perform these subgroup analyses due to insufficient data or potentially high probability of type I error induced by a large number of subgroup hypotheses [47]. Third, the result of the response to treatment suffered significant publication bias, which usually lead to an overestimation. Nevertheless, the adjusted analysis using the trim and fill method did not show substantial changes, suggesting that the publication bias should not significantly deviate the estimate.

## Conclusion

The current evidence suggests that using MTE as an adjuvant therapy to chemotherapy may improve the response to anticancer treatment and performance status in patients with gastric cancer. MTE may also reduce several chemotherapy adverse effects. Oral MTE may be a better choice. The reliability of these findings, however, is limited by the high risk of selection and performance bias across the included RCTs. Uncertainty remains regarding the effects of MTE on survival endpoints and the subgroup differences between acute and chronic use of MTE and between different chemotherapy regimens. Large-sample, long-term, double-blinded RCTs with reporting of any safety outcome are warranted to provide high-quality evidence on the efficacy and safety of MTE for treating gastric cancer.

## Data Availability

The datasets used and/or analyzed during the current study are available from the corresponding author on reasonable request.

## Abbreviations

CI: Confidence interval
CR: Complete response
FOLFOX: Folinic acid + fluorouracil + oxaliplatin
GRADE: Grading of Recommendations Assessment, Development and Evaluation; MTE *Marsdenia tenacissima* extract
OS: Overall survival
PD: Progressive disease
PFS: Progression-free survival
POR: Proportional odds ratio
PR: Partial response
PRISMA: Preferred Reporting Items for Systematic Reviews and Meta-Analyses; RCTs Randomized controlled trials
RECIST: Response Evaluation Criteria in Solid Tumors
RR: Risk ratio
SD: Stable Disease
XELOX: Capecitabine + oxaliplatin

## References

[1] Casamayor M, Morlock R, Maeda H, Ajani J (2018). Targeted literature review of the global burden of gastric cancer. Ecancermedicalscience.

[2] Chen W, Zheng R, Zhang S, Zeng H, Zou X, Hao J (2017). Report of cancer incidence and mortality in China, 2013. China Cancer.

[3] Japanese gastric cancer treatment guidelines 2014 (ver. 4). Gastric Cancer. 2017;20(1):1–19. 10.1007/s10120-016-0622-4 PMC5215069 27342689

[4] Sano T, Sasako M, Kinoshita T, Maruyama K (1993). China CancerRecurrence of early gastric cancer. Follow-up of 1475 patients and review of the Japanese literature. Cancer.

[5] Oba K, Paoletti X, Bang YJ, Bleiberg H, Burzykowski T, Fuse N (2013). Role of chemotherapy for advanced/recurrent gastric cancer: an individual-patient-data meta-analysis. Eur J Cancer.

[6] Wagner AD, Syn NL, Moehler M, Grothe W, Yong WP, Tai BC (2017). Chemotherapy for advanced gastric cancer. Cochrane Database Syst Rev.

[7] Xu W, Towers AD, Li P, Collet JP (2006). Traditional Chinese medicine in cancer care: perspectives and experiences of patients and professionals in China. Eur J Cancer Care (Engl).

[8] Farzaei MH, Bahramsoltani R, Rahimi R (2016). Phytochemicals as adjunctive with conventional anticancer therapies. Curr Pharm Des.

[9] Lan M. Medicinal plants in southern Yunnan (Chinese name: Dian Nan Ben Cao). Kunming: Yunnan People's Publishing House. 1436 (Ming Dynasty).

[10] Wang P, Yang J, Zhu Z, Zhang X (2018). Marsdenia tenacissima: a review of traditional uses, Phytochemistry and pharmacology. Am J Chin Med.

[11] Wang Q, Sun L, Xu J (2017). Correlation study on anti-tumor effect of marsdenia tenacissima and its preparation Xiaoaiping. Clin Misdiag Misther.

[12] Han SY, Ding HR, Zhao W, Teng F, Li PP (2014). Enhancement of gefitinib-induced growth inhibition by Marsdenia tenacissima extract in non-small cell lung cancer cells expressing wild or mutant EGFR. BMC Complement Altern Med.

[13] Liberati A, Altman DG, Tetzlaff J, Mulrow C, Gotzsche PC, Ioannidis JP (2009). The PRISMA statement for reporting systematic reviews and meta-analyses of studies that evaluate health care interventions: explanation and elaboration. Ann Intern Med.

[14] Schwartz LH, Seymour L, Litiere S, Ford R, Gwyther S, Mandrekar S (2016). RECIST 1.1 - standardisation and disease-specific adaptations: perspectives from the RECIST working group. Eur J Cancer.

[15] Mor V, Laliberte L, Morris JN, Wiemann M (1984). The Karnofsky performance status scale. An examination of its reliability and validity in a research setting. Cancer.

[16] Miller AB, Hoogstraten B, Staquet M, Winkler A (1981). Reporting results of cancer treatment. Cancer.

[17] Higgins JPT, Green S. Cochrane handbook for systematic reviews of interventions version 5.1.0; 2011. http://handbook.cochrane.orgJama.

[18] PROC GENMOD: Syntax: SAS/STAT(R) 9.3 User's Guide; 2011. https://support.sas.com/documentation/cdl/en/statug/63962/HTML/default/viewer.htm#genmod_toc.htm.

[19] Duval S, Tweedie R (2000). Trim and fill: a simple funnel-plot-based method of testing and adjusting for publication bias in meta-analysis. Biometrics.

[20] Deng W, Xu Y, Li N, Luo S (2016). Observation on clinical effects of Xiaoaiping injection combined with TP regimen in advanced gastric cancer. Chin J Geriaric Care.

[21] Gao L, Lu L, Hong C, Wang Y (2015). Analysis of Xiaoaiping injection combined with XELOX regimen in the treatment of advanced gastric cancer. Chin Arch Tradit Chin Med.

[22] Gao M, Liu L, An Z (2017). Observation on efficacy and adverse reactions of Xiaoaiping injection combined with chemotherapy in patients with advanced gastric cancer. Chin J Cancer Prev Treat.

[23] Huang J, Guo Y (2013). Inhibitory effects of Xiaoaiping tablet on acute adverse reactions during chemotherapy in patients undergoing radical gastrectomy. J Emerg Tradit Chin Med.

[24] Huo Y, Cheng G (2009). Clinical observation of xiaoaiping combined with chemotherapy for advanced gastric cancer. Chin Commun Doct.

[25] Keyoumu S, Ma L, Tang Y (2012). Clinical observation of Xiaoaiping injection combined with chemotherapy on the treatment of advanced gastric cancer. J Basic Clin Oncol.

[26] Li N, Ran J (2016). Clinical efficacy of Xiaoaiping combined with CPT-11 in the treatment of elderly patients with advanced gastric cancer. Hebei Med.

[27] Lin Q, Chen M, Xu X, Xu Z, Liu S, Zhou S (2015). Therapeutic effect of XELOX regimen combined with Xiaoaiping injection on advanced gastric cancer. Chin J Integr Tradit West Med Digest.

[28] Liu H, Zhu Z (2012). Study of Xiaoaiping injection combined with chemotherapy on treatment of advanced gastric cancer. Hebei Med.

[29] Liu W Clinical observation and study of Xiaoaiping injection combined with SOX regimen for advanced gastric carcinoma. Master [thesis] [硕士]. Shandong: Shandong University, 2017. Available from: China National Knowledge Infrastructure.

[30] Ma Y (2015). Therapeutic effect of Xiaoaiping injection combined with SOX regimen in treatment of advanced gastric cancer. World Clin Med.

[31] Shi W (2017). Role of Xiaoaiping tablet in controlling acute adverse reactions during chemotherapy in patients with gastric cancer. Guide China Med.

[32] Xiong L, Meng Y, Li D (2015). Effect of Xiaoaiping injection in patients with advanced gastric cancer chemotherapy. Shandong Med J.

[33] Zhang H, Song Z, Li B, Lu K, Wang X, Yin S (2015). Attenuated intervention of Xiao’aiping capsules in PF regimen chemotherapy for gastric cancer patients. Chin J Biochem Pharmaceut.

[34] Zhang H, Li X (2015). Efficacy of Xiaoaiping injection combined with XELOX regimen in the treatment of elderly patients with advanced gastric cancer. Jiangsu Med J.

[35] Zheng Z, Wang S, Song N (2017). Safety and efficacy of Xiaoaiping combined with docetaxel plus oxaliplatin in the second-line treatment of advanced gastric cancer. Chin J Hosp Pharm.

[36] Zhu D, Liang M, Yang M (2017). Xiaoaiping injection combined with FOLFOX6 chemotherapy in the treatment of advanced gastric cancer and the effect of intervention. Chin J Biochem Pharmaceut.

[37] Lin SS, Li FF, Sun L, Fan W, Gu M, Zhang LY (2016). Marsdenia tenacissima extract suppresses A549 cell migration through regulation of CCR5-CCL5 axis, rho C, and phosphorylated FAK. Chin J Nat Med.

[38] Davatgaran-Taghipour Y, Masoomzadeh S, Farzaei MH, Bahramsoltani R, Karimi-Soureh Z, Rahimi R (2017). Polyphenol nanoformulations for cancer therapy: experimental evidence and clinical perspective. Int J Nanomedicine.

[39] Wang X, Yan Y, Chen X, Zeng S, Qian L, Ren X (2018). The antitumor activities of Marsdenia tenacissima. Front Oncol.

[40] Hu Y, Wang S, He C, Liu T (2018). Research progress on anti-tumor properties of Marsdenia tenacissima. Tradit Med Res.

[41] Zeng ZP, Jiang JG (2010). Analysis of the adverse reactions induced by natural product-derived drugs. Br J Pharmacol.

[42] Porwal M, Khan NA, Maheshwari KK. Evaluation of Acute and Subacute Oral Toxicity Induced by Ethanolic Extract of Marsdenia tenacissima Leaves in Experimental Rats. Sci Pharm. 2017;85(3). 10.3390/scipharm85030029 PMC5620517 28825665

[43] Hatapakki BC, Hukkeri VI (2011). Antipyretic activity of root of Marsdenia tenacissima in rats. J Nat Remed.

[44] Zhu H, Liu F (2015). Analysis of ADR caused by Xiaoaiping injection in 13 cases. China Pharm.

[45] Lin X, Wang K, Xu W (2017). Reports on 46 cases of adverse drug reaction induced by traditional Chinese medicine injection. Eval Anal Drug-use Hosp China.

[46] Wu K, Zhu Z, He Y, Huang L, Yan X, Wang D (2019). Efficacy and safety of Xiao Ai ping injection combined with chemotherapy in advanced gastric Cancer: a systematic review and meta-analysis. Evid Based Complement Alternat Med.

[47] Sun X, Ioannidis JP, Agoritsas T, Alba AC, Guyatt G (2014). How to use a subgroup analysis: users' guide to the medical literature. JAMA.

[48] Whitehead A, Omar RZ, Higgins JP, Savaluny E, Turner RM, Thompson SG (2001). Meta-analysis of ordinal outcomes using individual patient data. Stat Med.

